# CCL11 released by GSDMD-mediated macrophage pyroptosis regulates angiogenesis after hindlimb ischemia

**DOI:** 10.1038/s41420-023-01764-9

**Published:** 2024-06-21

**Authors:** Yiwen Wang, Yang Gao, Huairui Shi, Rifeng Gao, Ji’e Yang, Ya’nan Qu, Shiyu Hu, Jian Zhang, Jingpu Wang, Jiatian Cao, Feng Zhang, Junbo Ge

**Affiliations:** 1 Department of Cardiology, Shanghai Institute of Cardiovascular Diseases, Zhongshan Hospital, Fudan University, 200032 Shanghai, China; 2 Department of Cardiology, Shanghai Fifth People’s Hospital, Fudan University, 200240 Shanghai, China; 3 Key Laboratory of Viral Heart Diseases, National Health Commission, 200032 Shanghai, China; 4 Key Laboratory of Viral Heart Diseases, Chinese Academy of Medical Sciences, 200032 Shanghai, China; 5 National Clinical Research Center for Interventional Medicine, 200032 Shanghai, China; 6 Institutes of Biomedical Sciences, Fudan University, 200032 Shanghai, China

**Keywords:** Cell death, Mechanisms of disease

## Abstract

Peripheral vascular disease (PVD) is an emerging public health burden with a high rate of disability and mortality. Gasdermin D (GSDMD) has been reported to exert pyroptosis and play a critical role in the pathophysiology of many cardiovascular diseases. We ought to determine the role of GSDMD in the regulation of perfusion recovery after hindlimb ischemia (HLI). Our study revealed that GSDMD-mediated pyroptosis occurred in HLI. GSDMD deletion aggravated perfusion recovery and angiogenesis in vitro and in vivo. However, how GSDMD regulates angiogenesis after ischemic injury remains unclear. We then found that GSDMD-mediated pyroptosis exerted the angiogenic capacity in macrophages rather than endothelial cells after HLI. GSDMD deletion led to a lower level of CCL11 in mice serum. GSDMD knockdown in macrophages downregulated the expression and decreased the releasing level of CCL11. Furthermore, recombinant CCL11 improved endothelial functions and angiogenesis, which was attenuated by CCL11 antibody. Taken together, these results demonstrate that GSDMD promotes angiogenesis by releasing CCL11, thereby improving blood flow perfusion recovery after hindlimb ischemic injury. Therefore, CCL11 may be a novel target for prevention and treatment of vascular ischemic diseases.

## Introduction

Peripheral vascular disease (PVD) is an increasingly serious public health problem, of which the disability and mortality rates are rising with the continuous increase of the aged population, the improving life quality, and the changing diet structure. Critical limb ischemia (CLI) is a kind of PVD with a high incidence rate, caused by tissue ischemia and hypoxia after arterial stenosis or occlusion. CLI results in circulatory disorders, which manifests as intermittent claudication, resting pain, ischemic ulcer, and other symptoms.

Conventional treatments of PVD such as angioplasty, stent implantation, and bypass surgery, have achieved success in treating local ischemic lesions. However, these methods are limited by their aggressiveness [1]. Insufficient neovascularization in ischemic regions is one of the main causes of perfusion impairment and limb dysfunction. Hence, promoting neovascularization is vital for perfusion and functional recovery based on the physiopathology of PVD, and is in urgent need of advanced development [2, 3]. Angiogenesis, the process of new capillaries generated from the originals, is necessary for perfusion and functional recovery in ischemic limbs of PVD patients. The procedure includes degradation of basal membrane, proliferation and migration of endothelial cells, endothelial reconstruction, and formation of new vascular networks [4].

Pyroptosis is a novel form of programmed cell death, which was identified after apoptosis and necrosis. Gasdermin D (GSDMD), a protein of Gasdermin family, is a hub molecule of pyroptosis process [5]. GSDMD has the ability to bind lipid and form protein pores on cell membranes, leading to plasma membrane rupture, cell swelling, release of intracellular substances including IL-1β and IL-18, and eventually cell death [6]. The involvement of GSDMD-mediated pyroptosis in cardiovascular diseases is well-documented [7–9]. However, the effects on endothelial function and angiogenesis remain controversial. It is reported that pyroptosis affects angiogenesis by releasing related inflammatory cytokines. IL-1β has been shown to promote angiogenesis in many studies [10–12], while the role of IL-18 remains double-sided. Inhibition of Nod-like receptor family pyrin domain-containing protein 3 (NLRP3) inflammasome activation and the release of IL-1β and IL-18 regulates endothelial cell proliferation and migration, further improving retinal neovascularization and alleviating leakage of impaired vessels [13]. On the contrary, a series of studies demonstrated the anti-angiogenic effect in tumor angiogenesis of IL-18 [14, 15]. Thus, there is considerable interest in clarifying the effect of GSDMD in angiogenesis and its underlying mechanisms.

In the present study, we used the GSDMD deficiency (Gsdmd^-/-^) transgenic mice to reveal the role of GSDMD in angiogenesis after hindlimb ischemia (HLI). Our research provided direct evidence that GSDMD-mediated pyroptosis regulated perfusion recovery after HLI by affecting the production and secretion of cytokines, aiming to identify novel targets in the pyroptosis process after ischemic injury for the prevention and treatment of PVD.

## Results

### GSDMD-mediated pyroptosis is activated in ischemic hindlimbs

**Fig. 1:**
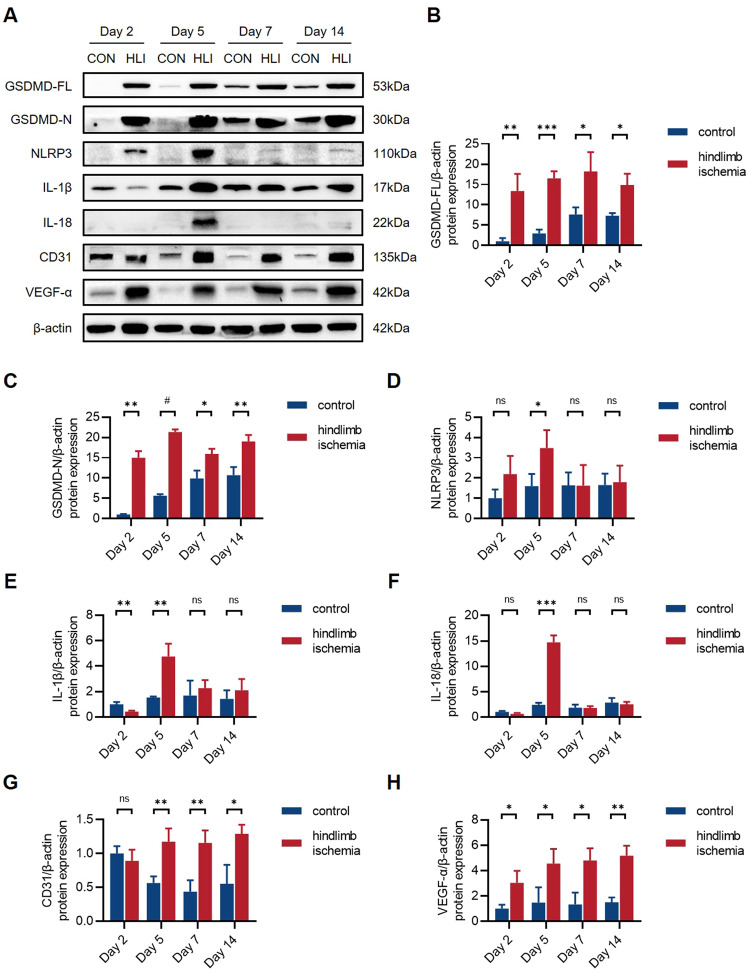
GSDMD-mediated pyroptosis is activated in ischemic hindlimbs. **A** Western blots and **B**–**H** quantitative analysis of GSDMD-FL, GSDMD-N, NLRP3, IL-1β, IL-18, CD31, and VEGF-α protein levels in ischemic gastrocnemius muscles of WT mice compared to non-ischemic controls at the 2nd, 5th, 7th, and 14th day post-HLI. *n* = 3 for each group; ns represents *p* > 0.05, *p < 0.05, **p < 0.01, ***p < 0.001, ^#^*p* < 0.0001.

To investigate the involvement of GSDMD-mediated pyroptosis in hindlimb ischemic injury in vivo, we established hindlimb ischemia (HLI) model by ligation of the femoral artery in wild-type (WT) mice. The protein expression levels of ischemic and non-ischemic gastrocnemius muscle tissues at different time points (the 2nd, 5th, 7th, and 14th day post-HLI) were determined by western blot analysis (Fig. 1A) (Supplementary 1). The results showed that the levels of full-length GSDMD (GSDMD-FL) and N-terminus of GSDMD (GSDMD-N) were significantly upregulated in ischemic tissues (Fig. 1B, C). Moreover, the levels of NLRP3, IL-1β, and IL-18 were also markedly upregulated after HLI, among which IL-1β and IL-18 markedly increased at the 5th day post-HLI, then decreased since the 7th day, indicating the inflammatory reaction peaked at the 5th day (Fig. 1D–F). Taken together, these results suggested that GSDMD-mediated pyroptosis might occur in gastrocnemius muscles after HLI. The higher levels of CD31 and VEGF-α in ischemic tissues indicated that angiogenesis occurs after ischemic injury, mediating the recovery of blood flow perfusion after HLI (Fig. 1G, H).

### Hypoxia stimulates pyroptosis in macrophages

**Fig. 2:**
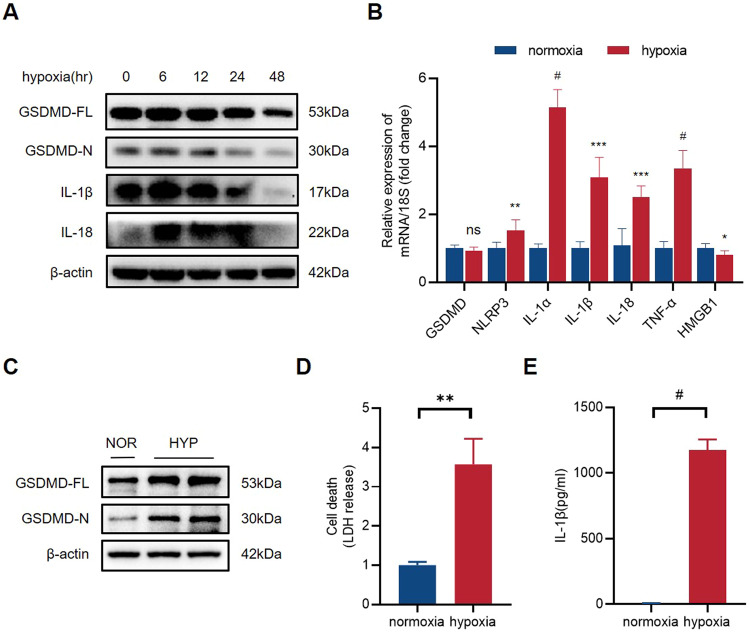
Hypoxia stimulates pyroptosis in macrophages. **A** Western blots of GSDMD-FL, GSDMD-N, IL-1β, and IL-18 protein expression levels in normoxia and hypoxia HUVECs. **B** The relative mRNA levels of GSDMD, NLRP3, IL-1α, IL-1β, IL-18, TNF-α, and HMGB1 of hypoxia THP-1 cells compared to normoxia THP-1 cells. **C** Western blots of GSDMD-FL and GSDMD-N protein expression levels in normoxia and hypoxia THP-1 cells. **D** The level of LDH in cell culture supernatants detected by LDH release test. **E** The level of IL-1β in cell culture supernatants detected by ELISA. *n* = 3 for each group; ns represents *p* > 0.05, *p < 0.05, **p < 0.01, ***p < 0.001, ^#^*p* < 0.0001.

Since both endothelial cells and macrophages participate in angiogenesis, we ought to determine the involvement of GSDMD in different cell types in the process. Therefore, we established a cellular hypoxia model, and normoxia as control to reveal whether hypoxia induces endothelial cell or macrophage pyroptosis in vitro. We extracted proteins of human umbilical vein endothelial cells (HUVECs) at different time points (6, 12, 24, and 48 h) after hypoxia and performed western blot analysis. The results showed that the levels of GSDMD-FL and GSDMD-N had no significant differences between normoxia and hypoxia endothelial cells (Fig. 2A) (Supplementary 1).

Then, a 24-h hypoxia intervention was also conducted on adherent human myeloid leukemia mononuclear cells (THP-1 cells) induced by Phorbol 12-Myristate 13-Acetate (PMA), a THP-1 cell differentiation inducer. The quantitative PCR was performed to verify the changes of cytokine mRNA levels in THP-1 cells after hypoxia. The results showed that the mRNA expression of NLRP3, IL-1α, IL-1β, IL-18, and TNF-α were significantly upregulated in hypoxia THP-1 cells (Fig. 2B). On the contrary to that in HUVECs, the protein levels of GSDMD-FL and GSDMD-N were significantly upregulated in hypoxia THP-1 cells (Fig. 2C) (Supplementary 1). We evaluated the degree of pyroptosis by measuring the LDH content in the cell supernatants of THP-1 cells. As we assumed, the LDH releasing level significantly increased after hypoxia in THP-1 cells (Fig. 2D). ELISA test demonstrated a higher level of IL-1β in hypoxia THP-1 cell culture supernatants (Fig. 2E). Collectively, hypoxia-induced GSDMD-mediated pyroptosis in macrophages.

### GSDMD deficiency inhibits perfusion recovery after HLI

**Fig. 3:**
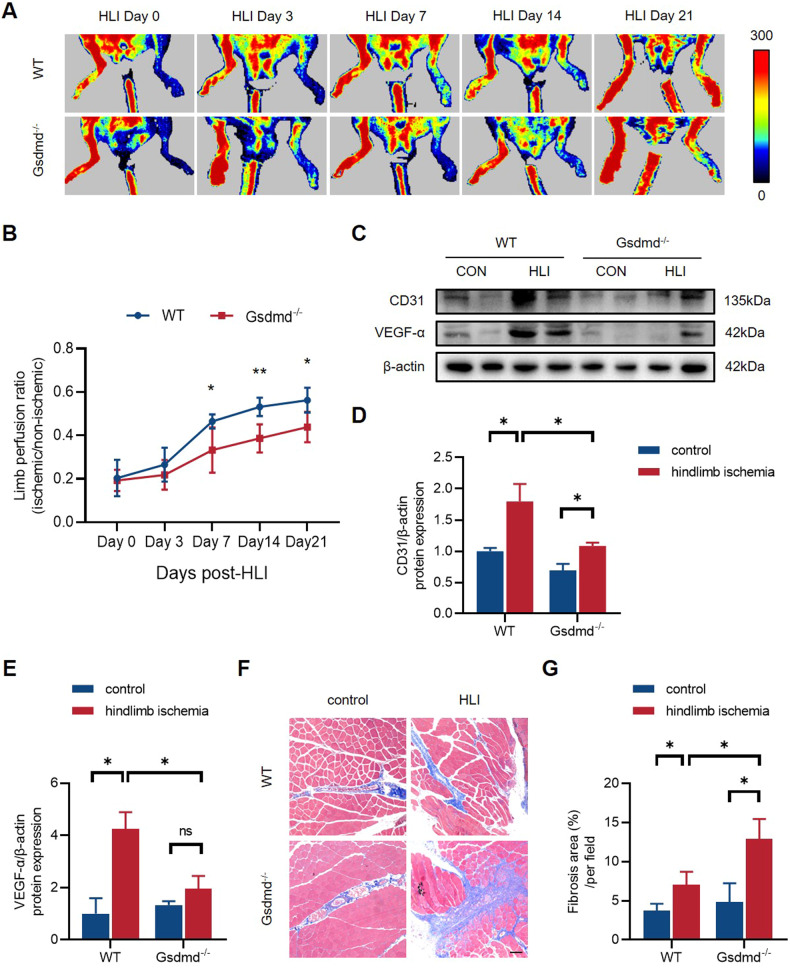
GSDMD deficiency inhibits perfusion recovery and angiogenesis in vivo after HLI. **A** Representative images of limb perfusion in WT and Gsdmd^-/-^ mice analyzed by Laser Doppler Perfusion Imaging and **B** quantitative Laser Doppler analysis measured by limb perfusion ratio of ischemic to non-ischemic hindlimbs (*n* = 5 for each group). **C** Western blots and **D**, **E** quantitative analysis of CD31 and VEGF-α expression levels of gastrocnemius tissues in WT and Gsdmd^-/-^ mice at 21st day post-HLI. **F** Representative images of Masson staining and **G** quantitative analysis of fibrosis area in gastrocnemius muscles in WT and Gsdmd^-/-^ mice at 21st day post-HLI. Fibrosis area was measured by Image Pro Plus. Scale bar = 100 μm; *n* = 3 for each group; ns represents *p* > 0.05, *p < 0.05, **p < 0.01, ***p < 0.001, ^#^*p* < 0.0001.

Next, we sought to unravel the contribution of GSDMD to blood flow perfusion recovery after HLI, owing to the upregulation of GSDMD in ischemic gastrocnemius muscles. We built models of HLI in GSDMD deficiency (Gsdmd^-/-^) mice and their litters (WT). Laser Doppler perfusion imaging of hindlimb blood flow was performed on the 3rd, 7th, 14th, and 21st day post-HLI to evaluate perfusion. The results, expressed in the form of perfusion ratio of ischemic to non-ischemic limb, showed that GSDMD^-/-^ mice group exhibited a lower blood flow perfusion recovery compared to WT mice group with significant differences at the 7th, 14th, and 21st day post-HLI (Fig. 3A, B). The gastrocnemius muscles of both ischemic and non-ischemic limbs were harvested on the 21st day post-HLI for subsequent analyses. Western blot results revealed a lower expression of CD31 and VEGF-α in the ischemic tissues of GSDMD^-/-^ mice compared to controls (Fig. 3C–E) (Supplementary 1), indicating that GSDMD deficiency decreased angiogenesis in vivo. Masson’s trichrome staining results showed that the fibrosis area of Gsdmd^-/-^ mice group was markedly larger than WT mice group (Fig. 3F, G), indicating a wider deposition of collagen fibers in the absence of GSDMD. Combining the results above, the lower blood flow perfusion recovery in Gsdmd^-/-^ mice group is attributed to decreased angiogenesis and aggravated tissue repairment caused by interstitial fibrosis after HLI.

### GSDMD overexpression and knockdown in endothelial cells do not affect endothelial function

**Fig. 4:**
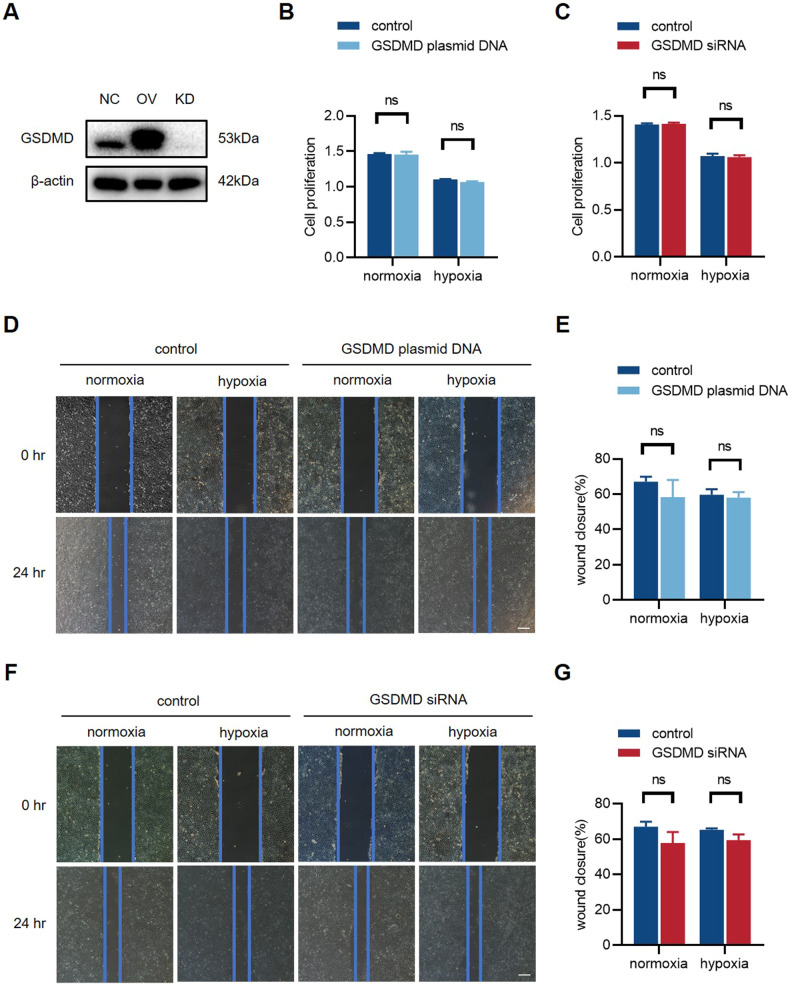
GSDMD overexpression and knockdown in endothelial cells do not affect endothelial function. **A** Western blot of GSDMD expression level of HUVECs transfected with plasmid DNA (GSDMD-OV) and siRNA (GSDMD-KD). **B**, **C** Relative cell proliferation rate of GSDMD-OV and GSDMD-KD HUVECs for 24 h. **D**, **E** Representative images and quantification of wound closure of GSDMD-OV and **F**, **G** GSDMD-KD HUVECs for 24 h. The value of relative wound closure was measured by ImageJ. Scale bar = 50 μm. *n* = 3 for each group; ns represents *p* > 0.05, *p < 0.05, **p < 0.01, ***p < 0.001, ^#^*p* < 0.0001.

To fully verify the effect of GSDMD and pyroptosis in endothelial cells, we generated GSDMD-overexpression (GSDMD-OV) and GSDMD-knockdown (GSDMD-KD) HUVECs by transfecting plasmid DNA and siRNA (Fig. 4A) (Supplementary 1). Next, we performed CCK-8 test. The results revealed that there was no significant difference in cell proliferation in GSDMD-OV HUVECs or GSDMD-KD HUVECs compared to controls (Fig. 4B, C). The wound healing assay also demonstrated that there was no significant difference in endothelial cell migration (Fig. 4D–G) after GSDMD overexpression or GSDMD knockdown.

### Inhibition of macrophage pyroptosis damages endothelial cell function

**Fig. 5:**
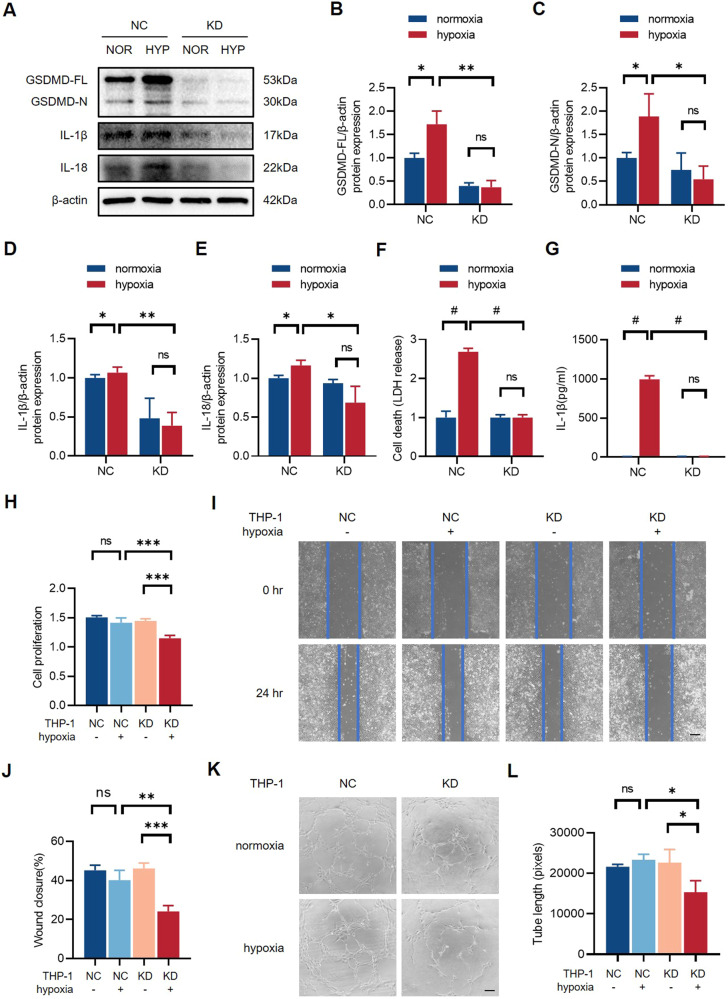
Inhibition of macrophage pyroptosis damages endothelial cell function. **A** Western blots and **B**–**E** quantitative analysis of GSDMD-FL, GSDMD-N, IL-1β, and IL-18 expression levels of GSDMD-NC and GSDMD-KD THP-1 cells after normoxia or hypoxia treatment. **F** The level of LDH in cell culture supernatants detected by LDH release test. **G** The level of IL-1β in cell culture supernatants detected by ELISA. **H** Relative cell proliferation rate of HUVECs stimulated with conditioned medium collected from normoxia/hypoxia GSDMD-NC and GSDMD-KD THP-1 cells for 24 h. **I**, **J** Representative images and quantification of wound closure for 24 h of HUVECs stimulated with the conditioned medium. **K**, **L** Tube formation assay and quantification in HUVECs stimulated with conditioned medium. The values of relative wound closure and tube length were measured by ImageJ. Scale bar = 50 μm. *n* = 3 for each group; ns represents *p* > 0.05, *p < 0.05, **p < 0.01, ***p < 0.001, ^#^*p* < 0.0001.

The contribution of macrophages to angiogenesis is well-documented. The observations above prompted us to confirm the role of GSDMD-mediated pyroptosis in macrophages. GSDMD-knockdown (GSDMD-KD) THP-1 cells were established and western blot results showed that the expression of GSDMD-FL, GSDMD-N, IL-1β, and IL-18 were significantly downregulated in GSDMD-KD THP-1 cells (Fig. 5A–E) (Supplementary 1). Meanwhile, GSDMD-KD THP-1 cells had a lower LDH releasing level after 24 h hypoxia than negative control (GSDMD-NC) THP-1 cells (Fig. 5F), and the secretion of IL-1β was also decreased in GSDMD-KD THP-1 cells (Fig. 5G), indicating that knockdown of GSDMD inhibited pyroptosis in macrophages. To further clarify whether macrophage pyroptosis affects endothelial function and angiogenesis, we generated a co-culture system of HUVECs and THP-1 cells. Specifically, the conditioned medium of GSDMD-NC and GSDMD-KD THP-1 cells underwent 24 h normoxia or hypoxia treatment was collected and used for HUVEC stimulation. HUVECs stimulated with condition medium were tested to illustrate endothelial function and angiogenic potential via CCK-8 kits, wound healing assay, and Matrigel tube formation assay. The results of CCK-8 test and wound healing assay indicated that HUVECs stimulated with conditioned medium from normoxia and hypoxia NC THP-1 cells had no significant differences in cell proliferation and migration. Meanwhile, HUVECs stimulated with hypoxia KD THP-1 cells exhibited impaired proliferation and migration compared to those stimulated with conditioned medium from normoxia KD THP-1 cells and normoxia/hypoxia NC THP-1 cells (Fig. 5H–J). In addition, HUVECs treated with the conditioned medium of NC group had a stronger angiogenic capacity (Fig. 5K–L). Therefore, we concluded that inhibition of GSDMD-mediated pyroptosis in macrophages weakened endothelial function and angiogenesis.

### GSDMD deficiency inhibits the expression and secretion level of CCL11 in vivo and in vitro

**Fig. 6:**
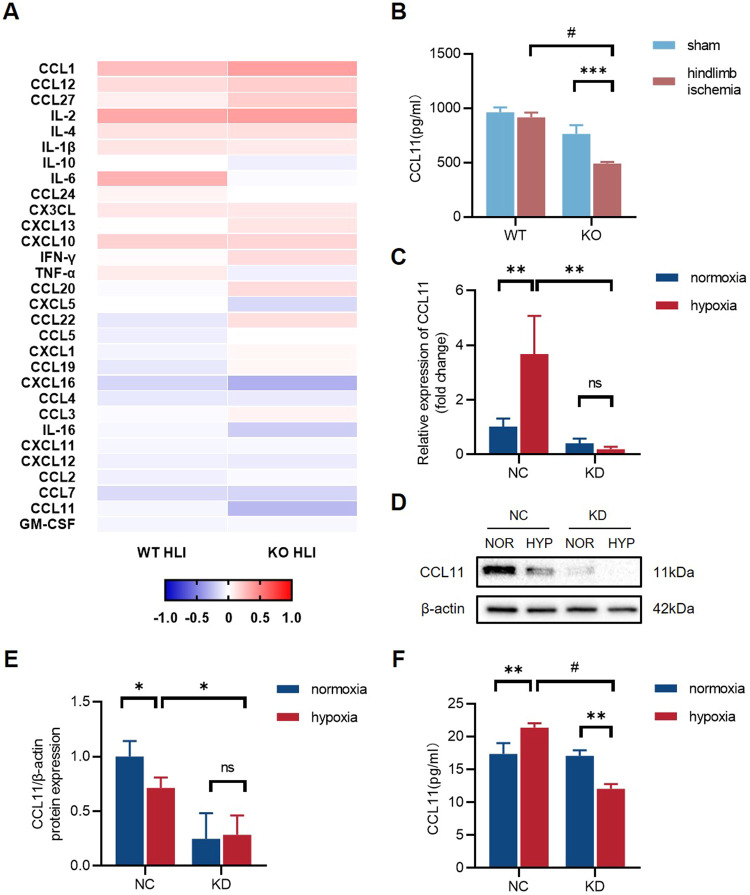
Inhibition of GSDMD-mediated pyroptosis leads to a decrease in the expression and secretion level of CCL11. **A** Heatmap for contrasting the mean log10 concentration values of cytokines for WT HLI and KO HLI mice serum. **B** The concentration of CCL11 in WT sham, WT HLI, KO sham, and KO HLI mice serum. **C** The relative mRNA level of CCL11 of GSDMD-NC and GSDMD-KD THP-1 cells after normoxia and hypoxia treatment. **D** Western blots and **E** quantitative analysis of CCL11 expression level of GSDMD-NC and GSDMD-KD THP-1 cells after normoxia and hypoxia treatment. **F** The level of CCL11 in cell culture supernatants detected by ELISA. *n* = 3 for each group; ns represents *p* > 0.05, *p < 0.05, **p < 0.01, ***p < 0.001, ^#^*p* < 0.0001.

In vitro observations verified that endothelial function and tube formation were exacerbated by the culture supernatants of GSDMD-KD macrophages. To demonstrate whether a specific cytokine plays a role in angiogenesis after hindlimb ischemia, we measured mice serum cytokine levels using Luminex technology in four groups: WT sham (*n* = 3), WT HLI (*n* = 4), KO sham (*n* = 3), and KO HLI (*n* = 4) to compare cytokine releasing levels between WT mice and GSDMD^-/-^ mice after HLI (Fig. 6A). Interestingly, we found a lower level of chemokine CC motif ligand 11 (CCL11) after HLI in Gsdmd^-/-^ mice serum than that in WT mice serum (Fig. 6B). We analyzed mRNA and protein expression of CCL11 in GSDMD-NC and GSDMD-KD THP-1 cells. The mRNA level of CCL11 in THP-1 cells was increased after hypoxia, whereas it decreased by the knockdown of GSDMD (Fig. 6C). Western blots results showed that CCL11 protein expression was downregulated in GSDMD-KD THP-1 cells (Fig. 6D, E) (Supplementary 1). Meanwhile, the concentration of CCL11 in culture supernatants was measured by ELISA. Results showed that CCL11 secretion level significantly increased after hypoxia in GSDMD-NC THP-1 cells and decreased after hypoxia in GSDMD-KD THP-1 cells (Fig. 6F). Collectively, these findings suggested that inhibition of pyroptosis in THP-1 cells may reduce the expression and release of CCL11.

### CCL11 facilitates endothelial cell proliferation, migration, and in vitro angiogenesis

**Fig. 7:**
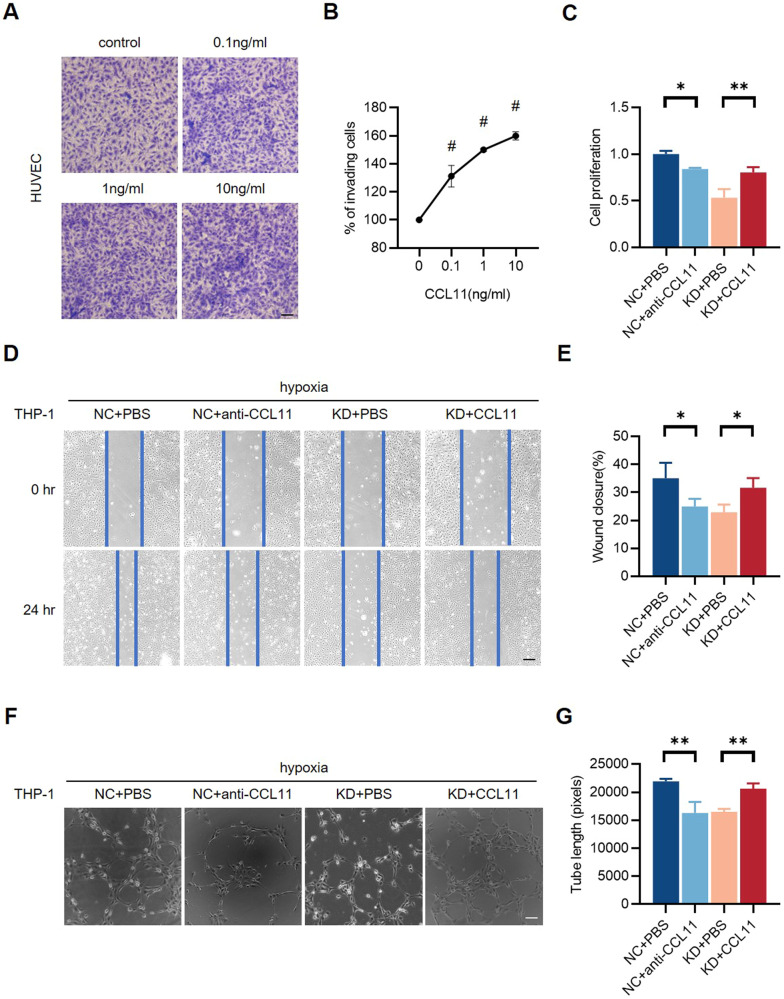
CCL11 facilitates endothelial cell proliferation, migration, and in vitro angiogenesis. **A**, **B** Representative images and quantification of chemotaxis of HUVECs toward CCL11 were assessed with transwell assay. There was a dose-dependent increase of chemotaxis toward CCL11. **C** Relative cell proliferation rate of HUVECs stimulated with conditioned medium collected from hypoxia THP-1 cells, CCL11, and CCL11 antibody (anti-CCL11). **D**, **E** Representative images and quantification of wound closure for 24 h of HUVECs stimulated with the conditioned medium, CCL11, and anti-CCL11. **F**, **G** Tube formation assay and quantification of tube length in HUVECs stimulated with the conditioned medium, CCL11, and anti-CCL11. The number of cells in transwell assay was counted by Image Pro Plus; the values of relative wound closure and tube length were measured by ImageJ. Scale bar = 50 μm. *n* = 3 for each group; ns represents *p* > 0.05, *p < 0.05, **p < 0.01, ***p < 0.001, ^#^*p* < 0.0001.

To verify whether the CCL11 released by pyroptotic THP-1 cells affects the endothelial function and angiogenesis in vitro, transwell assay was conducted to demonstrate the chemotaxis of HUVEC toward CCL11. The results showed that there was a dose-dependent migration of HUVECs toward CCL11, indicating that CCL11 released by THP-1 cells can affect endothelial cell function by its chemotaxin essence (Fig. 7A, B). To clarify the effect of CCL11 on angiogenesis and perfusion recovery, the co-cultural system established by HUVECs and hypoxia GSDMD-NC or GSDMD-KD THP-1 cells were further stimulated with recombinant human CCL11 (CCL11) or CCL11 antibody (anti-CCL11) to augment or neutralize the effect of CCL11. Cell function experiments were performed to assess endothelial function. CCK-8 assay showed that anti-CCL11 inhibited cell proliferation of HUVECs stimulated with conditioned medium from hypoxia GSDMD-NC THP-1 cells, while CCL11 repaired weakened cell proliferation of HUVECs stimulated with conditioned medium from hypoxia GSDMD-KD THP-1 cells (Fig. 7C). Wound healing assay revealed that anti-CCL11 aggravated endothelial cell migration induced by hypoxia GSDMD-NC THP-1, while CCL11 rescued impaired cell migration stimulated by hypoxia GSDMD-KD THP-1 cells (Fig. 7D, E). Matrigel tube formation assay showed that anti-CCL11 aggravated in vitro angiogenesis induced by hypoxia GSDMD-NC THP-1 cells, while CCL11 enhanced lower angiogenesis stimulated by hypoxia GSDMD-KD THP-1 cells, determining the pro-angiogenic effect of CCL11 (Fig. 7F, G). The results indicated that CCL11 secreted after 24 h hypoxia treatment facilitated endothelial cell proliferation, migration, and in vitro angiogenesis, while anti-CCL11 inhibited enhanced endothelial function.

## Discussion

In the present study, we provided direct evidence that GSDMD-FL and GSDMD-N were both upregulated in hindlimb ischemic injury. Meanwhile, the cleavage and activation of GSDMD were increased in hypoxia macrophages. The release of LDH, which is widely used as an auxiliary method to detect pyroptosis [16], also increased in the culture supernatants of hypoxia macrophages, indicating that pyroptosis mediated by GSDMD occurred after HLI. Furthermore, in the absence of GSDMD, mice exhibited a hindered perfusion recovery and an aggravated tissue repair after ischemia, which were associated with the inhibition of pyroptosis in injured tissues.

Injury repair and angiogenesis affect the recovery of blood flow perfusion after hindlimb ischemic injury. Macrophages and endothelial cells both play crucial roles in the process of angiogenesis [17]. In the early stage after injury, pro-inflammatory macrophages are mainly involved in the local inflammatory reaction, which promotes the recruitment of neutrophils by secreting pro-inflammatory cytokines. Neutrophils and macrophages then phagocytize the necrotic cell fragments and mediate the degradation of the basal membrane. In the late stage, anti-inflammatory macrophages constitute the majority of its population in injured tissues, inhibiting the inflammation and regulating cell functions of smooth muscle cells (SMC) and endothelial cells [18, 19] as well as the formation and reconstruction of new vascular networks [20–22].

Pyroptosis and the involvement of GSDMD were first found in macrophages [23, 24]. In recent years, studies have shown that pyroptosis can also be triggered by different stimulations in many other types of cells, such as endothelial cells [9, 25] and cardiomyocytes [7, 8]. Intriguingly, our study showed that GSDMD-mediated pyroptosis in endothelial cells had no significant effects on endothelial function and angiogenesis, while the inhibition of macrophage pyroptosis impaired endothelial function and in vitro angiogenesis, which was determined by the co-cultural incubation of THP-1 cells and HUVECs. Endothelial dysfunction and impaired angiogenesis stimulated by GSDMD-knockdown macrophage supernatants indicated that intracellular substances and cytokines released by pyroptotic macrophages stimulate endothelial cells and further regulate endothelial function and angiogenesis [26].

GSDMD-mediated pyroptosis exerts its biological effects by the release and activation of IL-1β and IL-18. IL-1β reportedly promotes angiogenesis, while the effect of IL-18 remains controversial. We observed that GSDMD deletion in mice led to aggravated perfusion recovery as well as downregulation of GSDMD in macrophages caused the inhibition of tube formation. Combining our findings and the evidence above, the effects of IL-1β and IL-18 cannot fully explain the decreased angiogenesis and perfusion recovery. To clarify the involvement of multiple cytokines, we here performed a Luminex analysis. The results showed a lower level of CC motif ligand 11 (CCL11) in the serum of GSDMD deficiency mice who underwent HLI, demonstrating that the effect of pyroptosis on angiogenesis and perfusion recovery after ischemia may be caused by CCL11.

CCL11, also known as Eotaxin, is a member of CC chemokine family with a CC domain on N-terminal. The biological effects of CCL11 are closely associated with its chemotaxis of recruiting eosinophils in inflammatory sites during allergic reactions [27, 28]. CCL11 can be produced by immune cells [29, 30] and a wide range of other cell types like epithelial cells, fibroblasts, and smooth muscle cells in allergic tissues [27, 31]. We observed that the releasing level of CCL11 decreased in the culture supernatants of THP-1 cells after GSDMD knockdown. Furthermore, mRNA and protein expression of CCL11 were both downregulated after GSDMD knockdown in THP-1 cells, suggesting that the absence of GSDMD not only reduced the release of CCL11 through pyroptosis process but also directly inhibited the CCL11 production. The formation of cell membrane pores and cell membrane rupture induced by GSDMD perform direct protein penetration across the plasma membrane, which is one of the important protein secretion pathway bypassing ER/Golgi routes for those lacking signal peptides [32]. In addition to IL-1β and IL-18, evidence shows that pyroptosis is also one of the secretory pathways of other cytokines, such as IL-33 [33, 34], IL-37 [35], TNF-α, and MCP-1(CCL2) [36]. Among these cytokines, although CCL2 contains a signal peptide, it can still be released through protein pores formed by GSDMD. CCL11 found in our study shares a similar structure with CCL2 in CC chemokine family. Therefore, we hypothesized that CCL11 can also be released by pyroptosis.

Besides the established role as an eosinophil-specific chemoattractant, CCL11 is also involved in cardiovascular diseases. CCL11 has an increased expression in cerebral aneurysms and abdominal aortic aneurysms (AAA), which indicates the potential usage as a biomarker in AAA and a possible therapeutic target for the prevention of aneurysm formation and rupture [37, 38]. Plasma and tissue inflammation are exacerbated by higher levels of CCL11 and other cytokines in the CD8a^+*tm1mak*^ mice (deficient in functional CD8 T-cells), leading to poor scar formation and the following cardiac rupture after myocardial infarction [39]. Besides, the levels of CCL11 and its main receptor CC motif receptor 3 (CCR3) are upregulated in human atherosclerosis, suggesting that CCL11 participates in vascular inflammation. CCL11 is highly expressed in SMC-rich area of the atherosclerosis plaque and injured arteries, suggesting that it plays an important role in regulating SMC migration [40, 41].

Although there is evidence supporting that migration and tube formation of lung endothelial cells induced by CCL11-CCR3 receptor-ligand pair contribute to airway remodeling in asthmatic lungs [42], the effect and underlying mechanisms of CCL11 on neovascularization after HLI remains unclear. In our study, we found that CCL11 induced in vivo angiogenesis. Recombinant human CCL11 improved the endothelial cell function including proliferation, migration, and tube formation, while the augmented endothelial function and angiogenic effect were neutralized by CCL11 antibody. Therefore, we concluded that GSDMD deficiency aggravated perfusion recovery, while CCL11 induced by GSDMD has a protective effect against perfusion recovery impairment by improving angiogenesis.

CCL11 is known to exert chemotactic activity by binding to CCR3 on eosinophils and induce the G protein-coupled receptor signaling. CCR3 is also identified on endothelial cells [42–44]. It is previously thought that CCL11 indirectly regulates angiogenesis which was dependent on eosinophil and mast cell products [45, 46]. However, there is evidence indicating that CCL11 induces CCR3-expressing angiogenesis in the absence of eosinophil infiltration [47]. Studies showed that CCR3 activation promotes human choroidal endothelial cell migration, proliferation and angiogenesis in the choroidal neovascularization, each of which (eotaxin-1/CCL11, eotaxin-2/CCL24, and eotaxin-3/CCL26) exerts the same effect through CCR3 [48]. Moreover, CCL11 mediates the paracellular permeability in human coronary artery endothelial cells through activation of the p38 MAPK, Stat3 and NF-κB signaling pathways [49]. CCL11 induces choroidal endothelial cell migration by activating CCR3 through PI3K/Rac1 signaling [50].

Our current results demonstrated that CCL11 exerted chemotaxis of HUVECs in a dose-dependent manner and the angiogenic capacity proved by recombinant human CCL11 and CCL11 antibody. Combined with the evidence above, we concluded that CCL11 can act as a direct mediator of angiogenesis in hypoxia conditions. Nevertheless, the effect of CCL11 in vivo is still undefined. The supplement of CCL11 in GSDMD deficiency mice or transgenic animals with CCL11 deficiency and the observation of following perfusion recovery after HLI is essential in subsequent research.

In summary, our study indicates that GSDMD-mediated pyroptosis promotes perfusion recovery of ischemic hindlimbs by regulating the expression and secretion of CCL11 in macrophages. We provide a new understanding of the biological functions of CCL11 in the vascular system. Based on these findings, supplements of chemokines CCL11 may be a new therapeutic strategy for inducing neovascularization and improving perfusion recovery, thereby preventing the occurrence of adverse events such as disability and death in peripheral vascular diseases.

## Materials and methods

### Reagents and antibodies

The following antibodies were used for western blotting: anti-NLRP3(15101S, Cell Signaling Technology), anti-GSDMD (NBP2-33422, Novus Biologicals), anti-IL-1β(ab234437, Abcam), anti-IL-18 (ab207323, Abcam), anti-CD31(AP3628, R&D systems), anti-VEGF (sc-7269, Santa Cruz), anti-CCL11(ab133604, Abcam), anti-β-actin (4970S, Cell Signaling Technology). Recombinant human Eotaxin/CCL11 (300-21, PeproTech) and Eotaxin/CCL11 monoclonal antibody (MAB320, R&D systems) for neutralization were used in cell experiments.

### Animals

Wild-type (WT) C57BL/6J mice were purchased from Jie Si Jie Laboratory Animal Corp (Shanghai, China). GSDMD deficiency (Gsdmd^-/-^) mice from Cyagen Biosciences Inc. (Guangzhou, China) were used in this study. All animals were maintained at SPF animal care facility of Zhongshan Hospital, Fudan University according to the National Institutes of Health Guidelines for the Care and Use of Laboratory Animals, and the experimental protocols were approved by the Animal Care and Use Committee of Zhongshan Hospital, Fudan University.

### Hindlimb ischemia models

Male mice aged 8 weeks and weighing 22–28 g were used in the experiment. Mice were anesthetized with 1% pentobarbital according to their body weight (50 mg/kg), and both sides of the hindlimb were depilated. A longitudinal skin incision and passive separation of connective tissue and fat tissue were made to expose the femoral vessels. The left femoral artery was separated from the vein and nerve, ligated, and excised. After ligation, the skin was closed and sterilized. Laser Doppler Perfusion Imaging was used to detect the blood flow perfusion. The operation was successful while the perfusion ratio of the ischemic side to the non-ischemic side is less than 0.2.

### Laser Doppler perfusion imaging

The mice were anesthetized with 1% pentobarbital, the hindlimb were completely depilated. A Laser Doppler Perfusion Imaging analyzer, PeriScan PIM 3 (Perimed, Sweden), was used to measure blood flow of the hindlimb at the 3rd, 7th, 14th, and 21st days post-HLI. PIMSOFT software was used to select the ischemic area of ischemic limb and non-ischemic limb to measure the ROI value, and the results were expressed in the form of perfusion ratio of ischemic to non-ischemic limb.

### Masson’s trichrome analysis

Gastrocnemius muscles from the ischemic limbs were harvested at the 3rd, 7th, 14th, and 21st days after HLI surgery and fixed in phosphate-buffered formalin, embedded in paraffin. The entire length of the muscle was sectioned and followed by Masson’s trichrome staining for the observation of muscle fibrosis.

### Western blot assay

Gastrocnemius muscles were collected for the detection of NLRP3, GSDMD-FL, GSDMD-N, IL-1β, IL-18, CD31, and VEGF-α, while HUVECs and THP-1 cells were collected for the detection of GSDMD-FL, GSDMD-N, IL-1β, IL-18, and CCL11. Tissues and cells were lysed in RIPA lysis buffer containing 1% protease inhibitor, phosphatase inhibitor, and PMSF for 30 min at 4 °C, followed by centrifugation at 12,000 rpm for 15 min. The supernatants were collected and mixed with 5× loading buffer. The isolated proteins were separated by SDS-PAGE and then transferred onto PVDF membranes. The membranes were blocked with 5% BSA-TBST (5% BSA and 1% Tween-20). Immunoblots were incubated overnight at 4 °C with primary antibodies, followed by HRP-conjugated secondary antibodies of appropriated species for 1 h at room temperature. After washing in TBST, the detection of immunocomplexes was performed on an automatic chemiluminescence imaging analysis system (Bio-Rad, CA USA) and Image Lab software (3.0, Bio-Rad) at enhanced chemiluminescence.

### RNA extraction and quantitative RT-PCT (qRT-PCR)

Total RNA was extracted using Trizol reagent (Invitrogen, Carlsbad, USA) according to the standard RNA isolation protocol. The concentration of RNA was measured by NanoDrop 8000 spectrophotometer (Thermo Fisher Scientific, USA). Single-stranded complementary DNA was synthesized from 200 ng RNA in a 20 μl reaction volume with reverse transcription kits (Takara, Japan), and the reaction was performed according to the manufacturer’s protocol. qRT-PCR primers were selected according to the species sequence on PrimerBank and synthesized by Tsingke Biotechnology Co., Ltd. (Beijing, China). The primers used in this study were shown in Table 1. qRT-PCR was carried out using a SYBR Green PCR kit (Yeasen Biotechnology, Shanghai) following the protocol provided by the manufacturer and the cycle threshold (CT) of each gene was recorded. The 18S was used as an internal reference to calculate mRNA expression. Data were analyzed by the comparative CT method (2^−ΔΔCt^).

**Table 1:** qRT-PCR primer sequences.

Gene symbol	Polarity	Sequence
GSDMD	Forward	GTGTGTCAACCTGTCTATCAAGG
	Reverse	CATGGCATCGTAGAAGTGGAAG
NLRP3	Forward	GATCTTCGCTGCGATCAACAG
	Reverse	CGTGCATTATCTGAACCCCAC
IL-1α	Forward	TGGTAGTAGCAACCAACGGGA
	Reverse	ACTTTGATTGAGGGCGTCATTC
IL-1β	Forward	TTCGACACATGGGATAACGAGG
	Reverse	TTTTTGCTGTGAGTCCCGGAG
IL-18	Forward	TCTTCATTGACCAAGGAAATCGG
	Reverse	TCCGGGGTGCATTATCTCTAC
TNF-α	Forward	GAGGCCAAGCCCTGGTATG
	Reverse	CGGGCCGATTGATCTCAGC
HMGB1	Forward	TATGGCAAAAGCGGACAAGG
	Reverse	CTTCGCAACATCACCAATGGA
CCL11	Forward	CCCCTTCAGCGACTAGAGAG
	Reverse	TCTTGGGGTCGGCACAGAT
18S	Forward	GCAATTATTCCCCATGAACG
	Reverse	GGCCTCACTAAACCATCCAA

### Cell culture

Primary human umbilical vein endothelial cells (HUVECs) were cultured in Endothelial Cell Medium (ECM) supplemented with 5% fetal bovine serum (FBS) and 1% endothelial cell growth supplement (ECGS). THP-1 cells, a human myeloid leukemia mononuclear cell line, were cultured in RPMI-1640 medium with 10% FBS, and 25 ng/ml PMA for induction of differentiation from monocytes to macrophages for 24–48 h before use. All the cells were incubated at 37 °C in 5% CO_2_.

### Cell transfection

When HUVECs confluency met approximately 60–70%, cells in 6-well plates were transfected with plasmid DNA or siRNA for overexpression or knockdown of GSDMD using Lipofectamine^TM^ 3000 (Invitrogen). Plasmid DNA and siRNA were synthesized by OBiO Technology (Shanghai, China). After 8 h of transfection, the medium was changed by fresh ECM containing 5% FBS and cells were harvested or used for further study in vitro after 48 h. siRNAs specifically targeting GSDMD and a non-targeting control siRNA were designed and synthesized by Genechem (Shanghai, China). The siRNA sequences were as follows: siRNA-GSDMD-1: 5′-GCCAGAACACAAAGTCCTGCA-3′, siRNA-GSDMD-2: 5′-GAGCTTCCACTTCTACGATGC-3′, siRNA-GSDMD-3: 5′-GCTGGTTATTGACTCTGACTT-3′, nonsense siRNA: 5′-TTCTCCGAACGTGTCACGT-3′. The siRNA sequences were inserted into the GV112 lentivirus vector containing a gcGFP fluorescent marker and sequenced to confirm the correct identity of the siRNAs. The plasmid carrying the target gene (GV) and virus packaging helper plasmids (pHelper 1.0 and pHelper 2.0) were purified and transfected into monolayer 293T cells cultured in DMEM with 10% FBS for 48 h. The lentivirus was purified from the supernatant of the transfected 293T cells by centrifugation. The viral supernatant was transfected into THP-1 cells. Stable THP-1 cell lines with knockdown of GSDMD were established by puromycin (5 μg/ml) screening for further study.

### Co-culture of HUVECs and THP-1 cells

When the HUVEC confluency met approximately 60–70%, the conditioned medium collected from THP-1 cell supernatants stimulated by hypoxia intervention and ECM was added into 6-well plates at a ratio of 1:1 and incubated for 24 h to simulate cell co-culture. Hypoxia cells were incubated in 5% CO_2_ and 95% N_2_ for 24 h.

### Enzyme-linked immunosorbent assay (ELISA)

After normoxia or hypoxia incubation for 24 h, the culture medium of the adherent THP-1 cells was collected and followed by centrifugation at 1000 rpm for 10 min. The supernatants were separated and stored at −80 °C before use. The assay was performed according to the protocol provided by the manufacturer, and the IL-1β and CCL11 levels were calculated by the standard curve. Human IL-1β ELISA Kit (RK00001, Abclonal, China) and Human Eotaxin/CCL11 ELISA kit (ELH-Eotaxin-1, Raybiotech, China) were used for analysis.

### Lactate dehydrogenase release assay

Lactate Dehydrogenase Release Assay Kit (C0016, Beyotime Biotechnology, China) was used to detect the level of LDH in cell culture supernatants. The THP-1 cells were seeded in 6-well plates and the culture supernatants were collected after 24 h normoxia or hypoxia intervention. The assay was performed according to the protocol provided by the manufacturer. The LDH level were calculated by the standard curve.

### CCK-8 cell proliferation assay

Cell Counting Kit 8 (C0037, Beyotime Biotechnology) was used to obtain cell viability. 2 × 10^3^ cells per well were seeded in 96-well plates. All these cells were cultured for 24 h. Then we discard the culture medium and add 100 μl CCK-8 solution (diluted with ECM to 10% concentration) into each well, and incubated the 96-well plates for 2 h, and the absorbance of the cells at 450 nm was measured by the multifunctional reader FlexStation 3 (Molecular Devices, CA, USA).

### Wound healing assay

Three horizontal lines were drawn on the back of 6-well plates, and then 5 × 10^5^ cells per well were seeded in plates. After overnight incubation to ensure the cells grew fully, the cell monolayer in each well was scratched using a plastic tip vertically across the plate and then washed twice with PBS. Subsequently, the cells were incubated in the serum-free medium with different treatments. Images were taken at 0 and 24 h to measure the distance of the wound.

### In vitro matrigel tube formation assay

We use the Matrigel tube formation assay to quantify in vitro angiogenesis. 10 μl Matrigel (356234, Corning, NY, USA) per well was vertically added into the angiogenesis slide (81506, ibidi, Germany). Then, HUVECs were trypsinized and resuspended in the conditioned medium collected from different treated THP-1 cells at 2 × 10^5^ cells/ml. We seeded 50 μl of cell suspension into the angiogenesis slide, followed by a 6 h incubation at 37 °C in 5% CO_2_. The number of total tube lengths per field was quantified by ImageJ software (1.52a, NIH, USA).

### Luminex analysis

Blood samples were obtained at 3 days after HLI from WT and GSDMD^-/-^ mice. A multiplex bead-based assay was developed using Luminex Flowmetrix system X-200 (Luminex, TX, USA) to investigate the cytokines levels in the serum. Test sera from mice were examined with multiplex assays (LX-MultiDTH-27, Bio-Rad) for several cytokines. First, plates with beads were added with 50 μl diluted standards or serum samples and then incubated for 30 min at room temperature (RT). Next, mixtures were incubated with biotin-conjugated antibodies 25 μl per well for 30 min at RT, followed by incubation for 30 min with phycoerythrin-conjugated streptavidin. Finally, the concentration of cytokines in the bead array was measured with the fluorescence intensity calculated with the Flowmetrix software.

### Transwell assay

A quantity of 2 × 10^4^ endothelial cells in 200 μl serum-free ECM medium was added to the upper transwell chamber (3422, Corning), with the lower chamber filled with 600 μl ECM containing 20% FBS. Meanwhile, 0.1, 1, 10 ng/ml CCL11 were added to cells, which were cultured for 48 h at 37 °C. After incubation, cells remaining on the top of the transwell chamber were removed. Migrated cells on the bottom of the filter were fixed in 4% paraformaldehyde and stained with 0.1% crystal violet and the number of migrated cells in each well was counted using light microscopy. Each group had three independent duplications.

### Statistical analysis

ImageJ 1.44P was used for quantitative analysis of immunoblotting, and GraphPad Prism 8.0 (GraphPad Software, CA, USA) was used for data statistics and mapping. *T*-test or single factor analysis of variance (ANOVA) was used to compare parametric variables between groups. All data are presented as mean ± SEM. The value of *p* < 0.05 was considered statistically significant. In the statistical result chart, ns represents *p* ≥ 0.05, *p < 0.05, **p < 0.01, ***p < 0.001, and ^#^*p* < 0.0001.

### Supplementary information

Original Data File

Original Data File

Original Data File

Supplementary material 1

## Data Availability

The data supporting the findings of the study are available from the corresponding author upon reasonable request.
