# Supplementary figures and images for: CCL11 released by GSDMD-mediated macrophage pyroptosis regulates angiogenesis after hindlimb ischemia
Source: Cell Death Discov. 2024 Jun 21;10:294. doi: 10.1038/s41420-023-01764-9 (PMC11192718; doi:10.1038/s41420-023-01764-9)

Figure1A

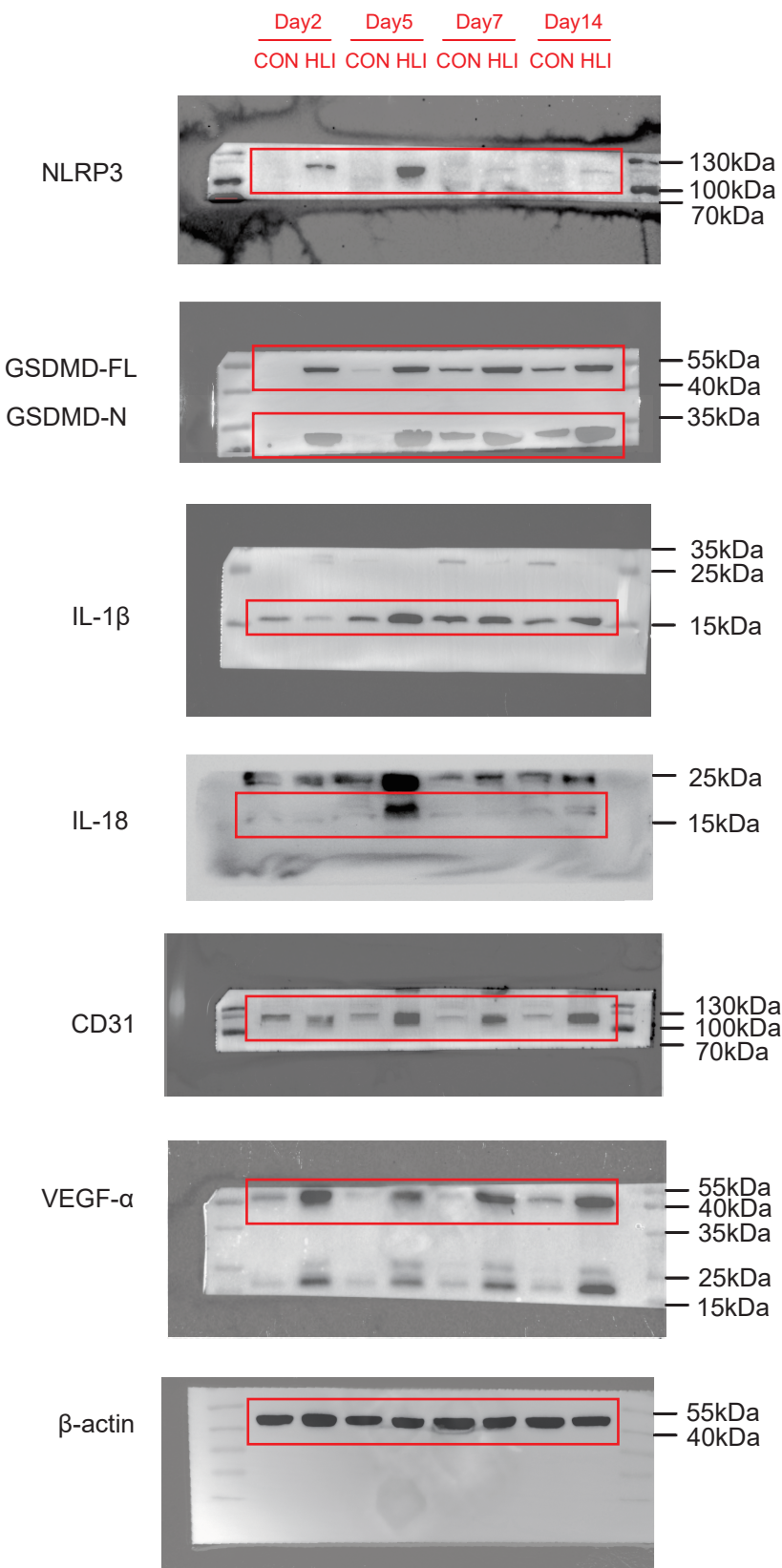

Figure 2A

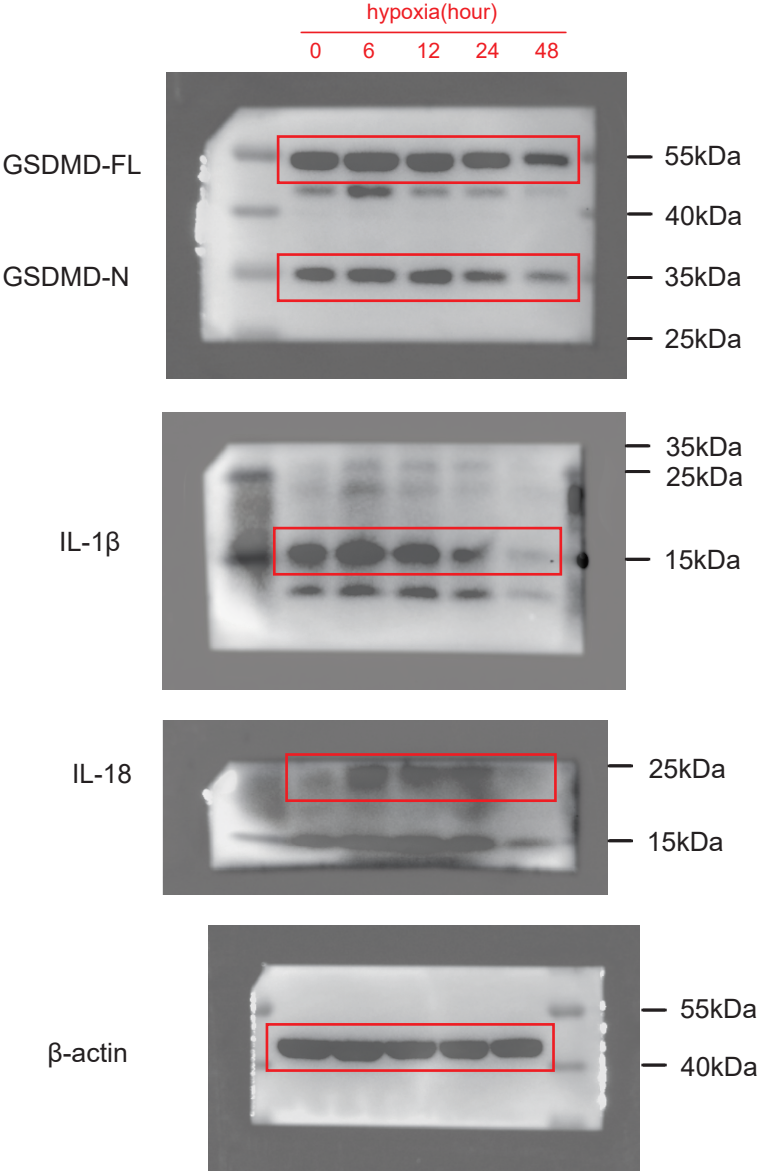

Figure 2C

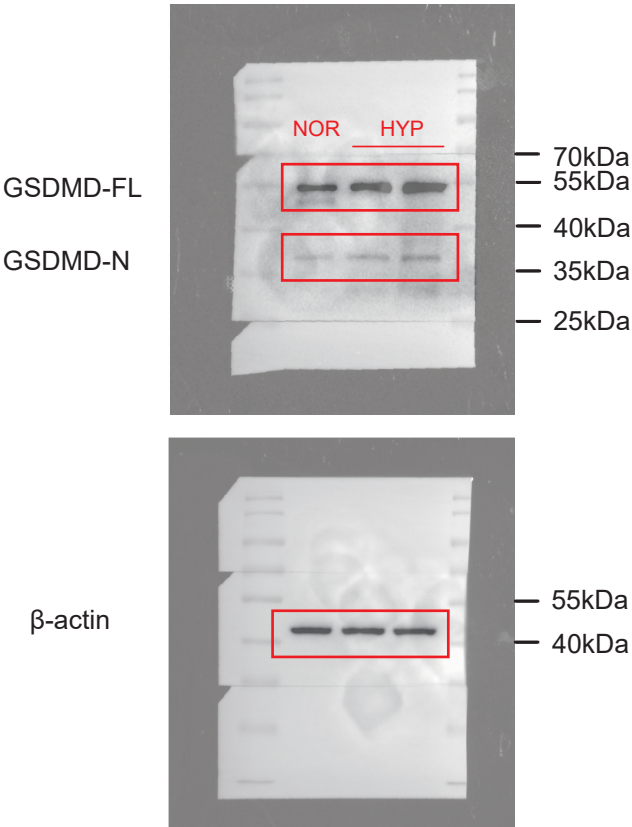

Figure 3C

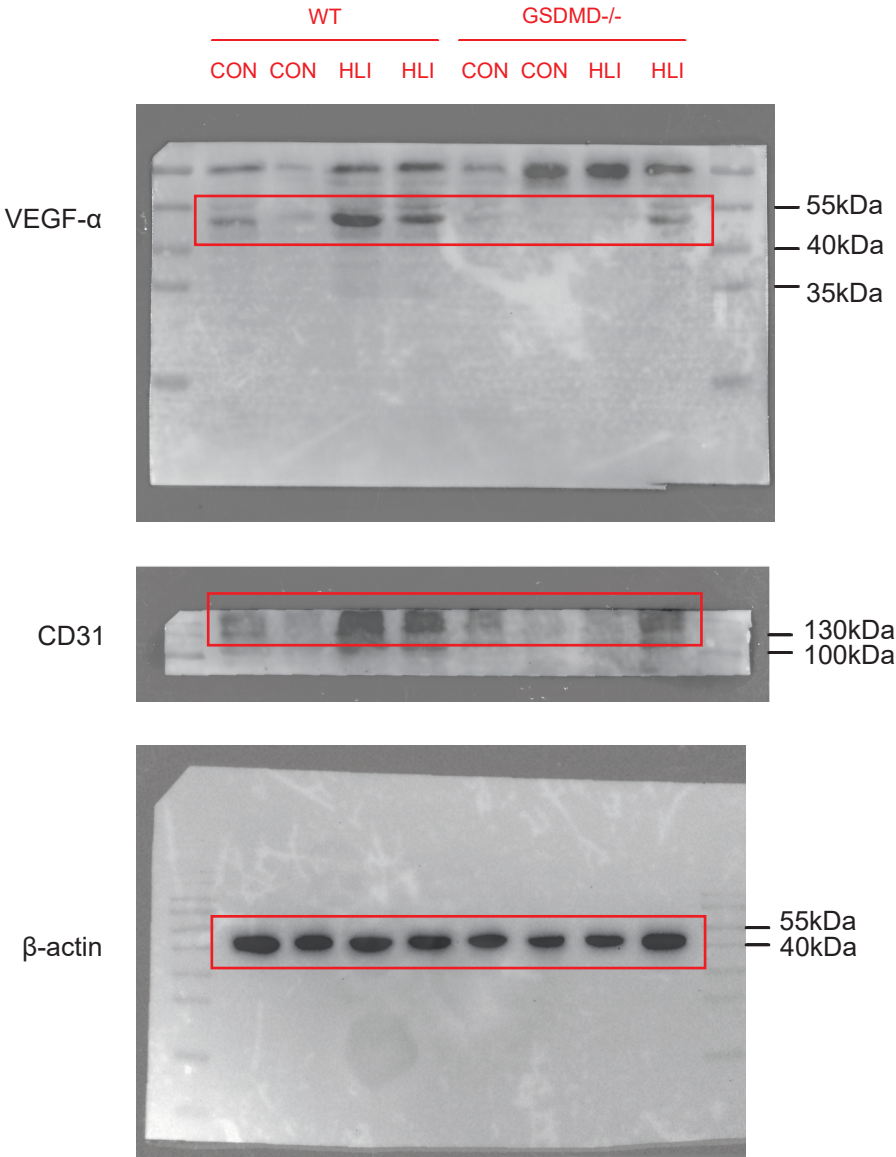

Figure 4A

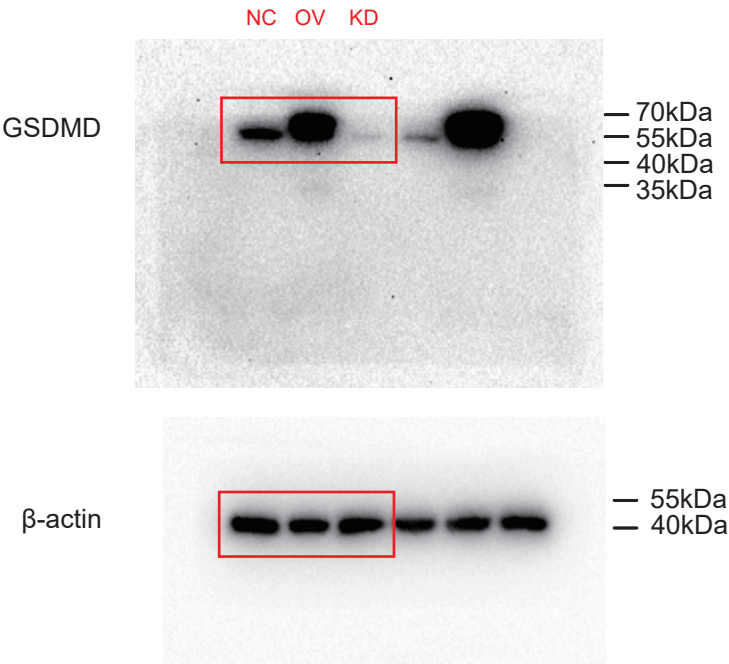

Figure 5A

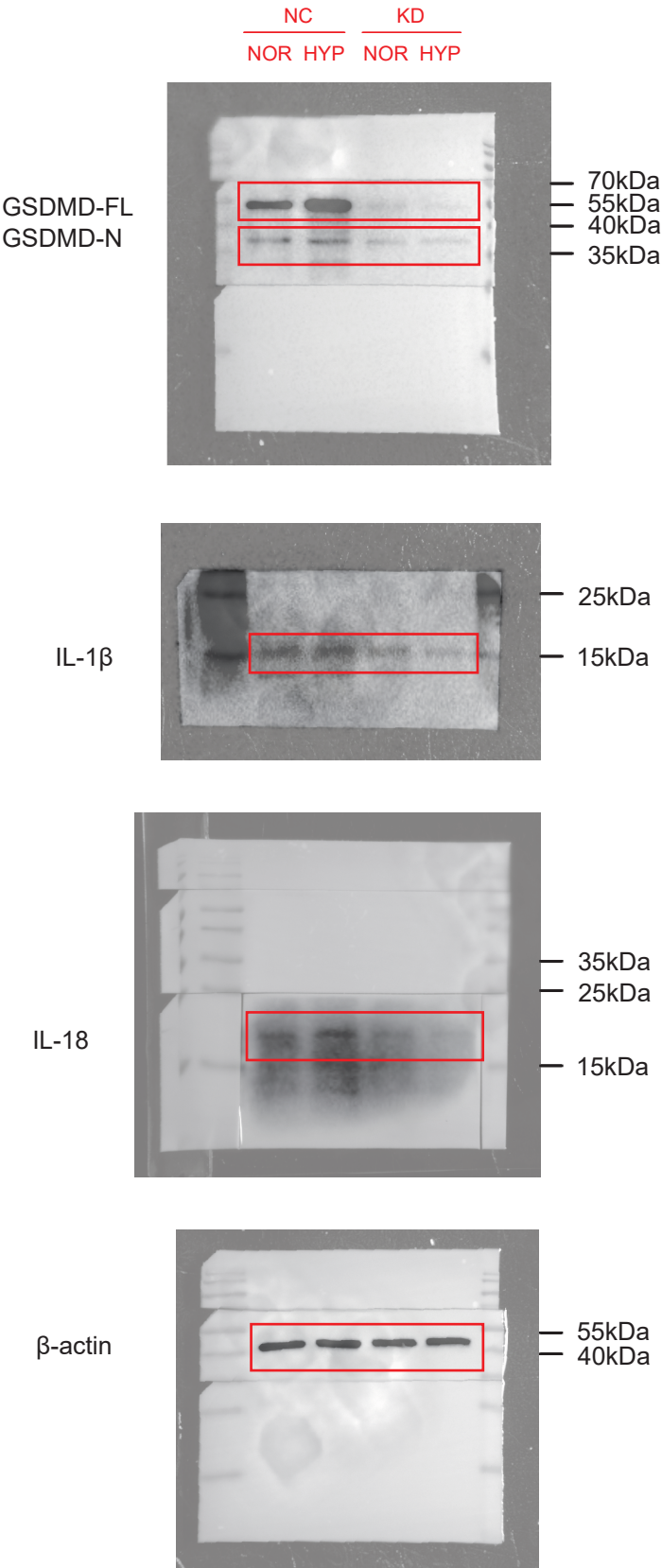

Figure 6D

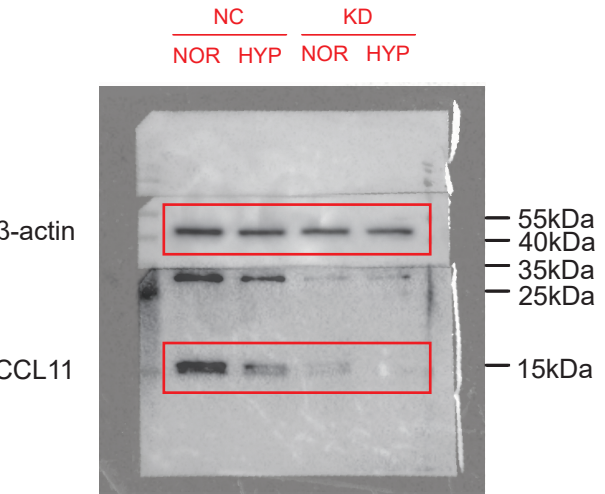

Supplement: Supplementary file 3 — Original Data File [file 41420_2023_1764_MOESM3_ESM.pdf]

Figure1A

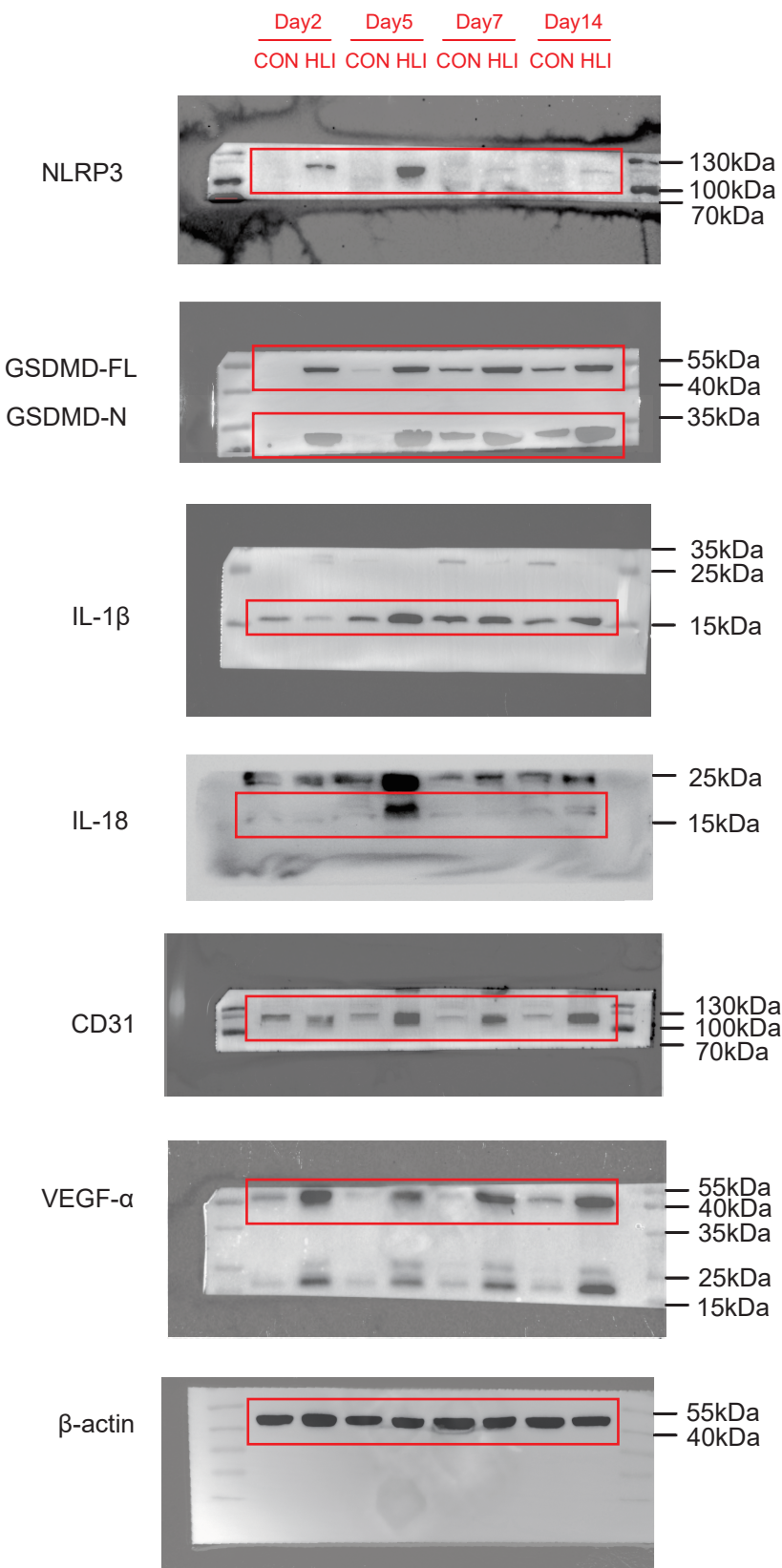

Figure 2A

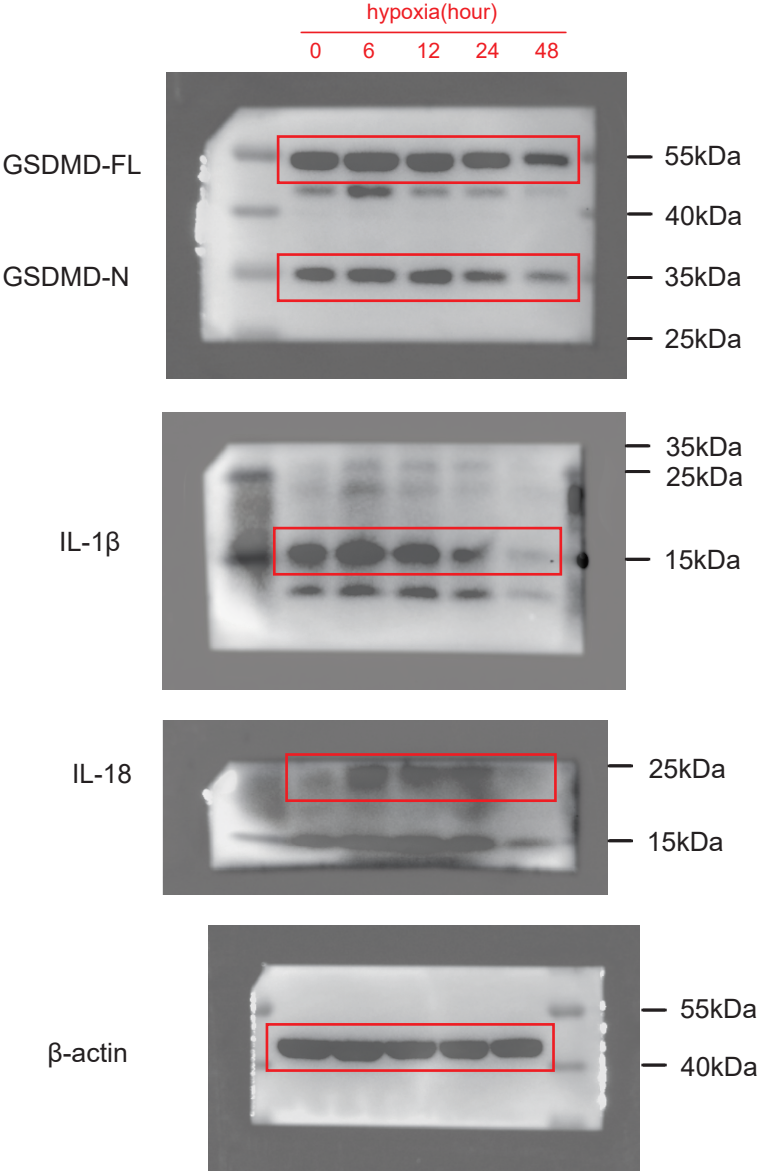

Figure 2C

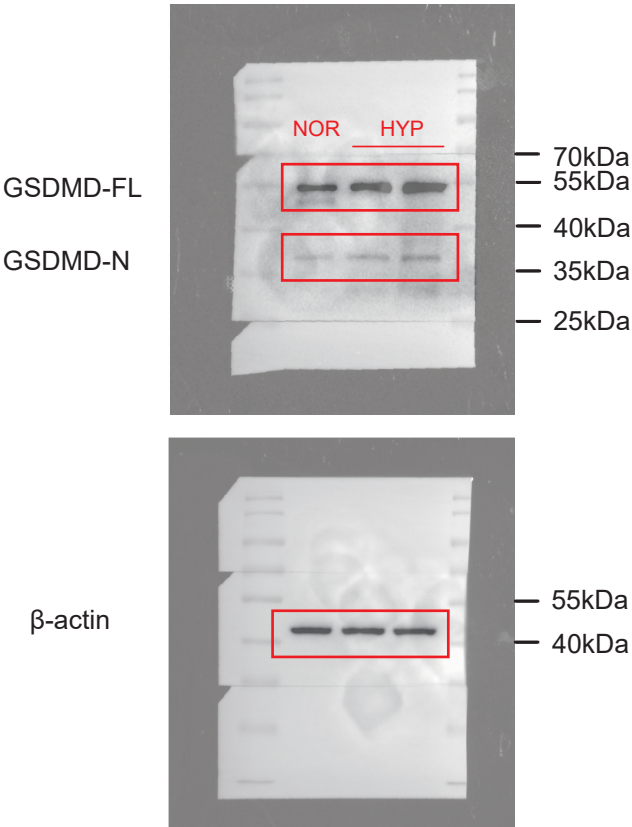

Figure 3C

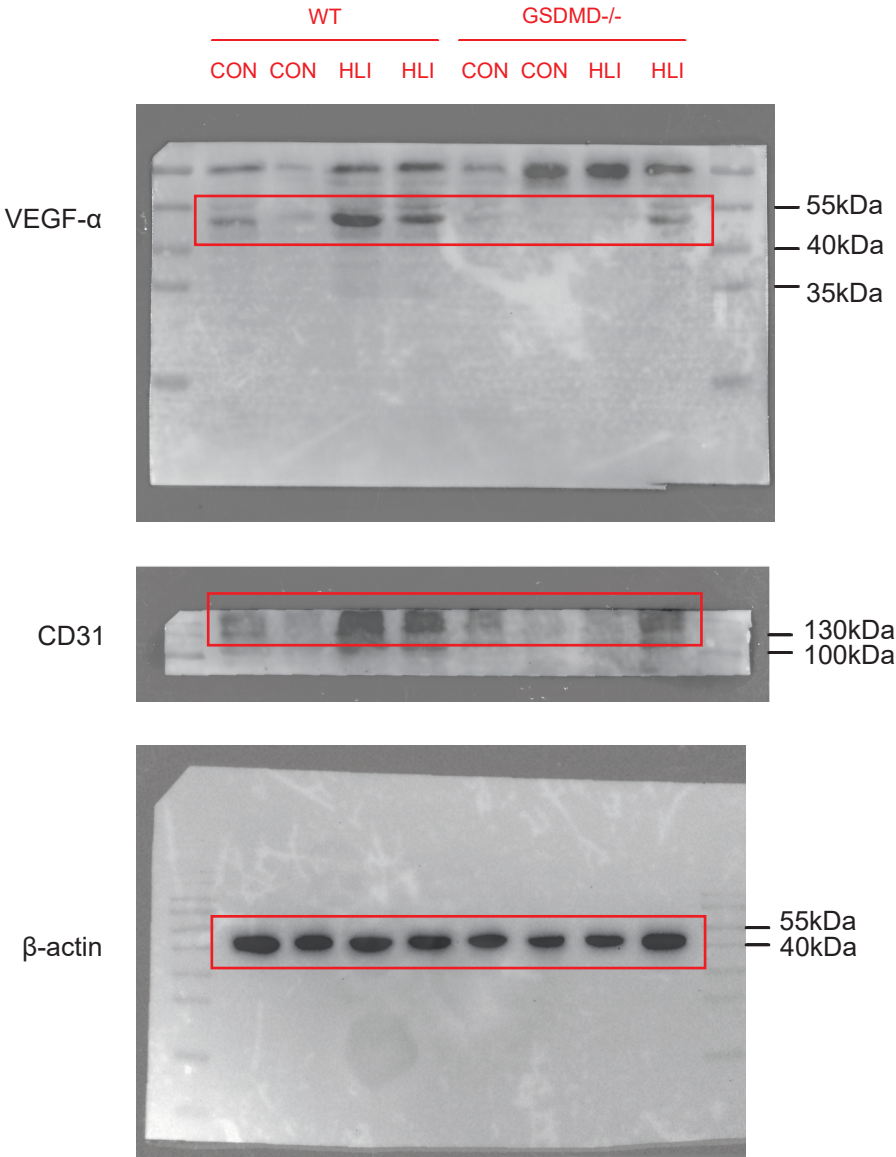

Figure 4A

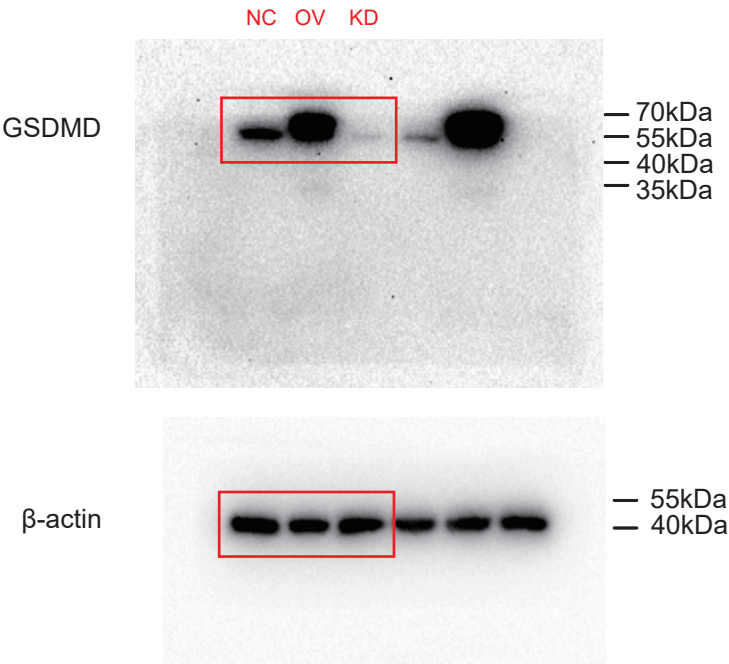

Figure 5A

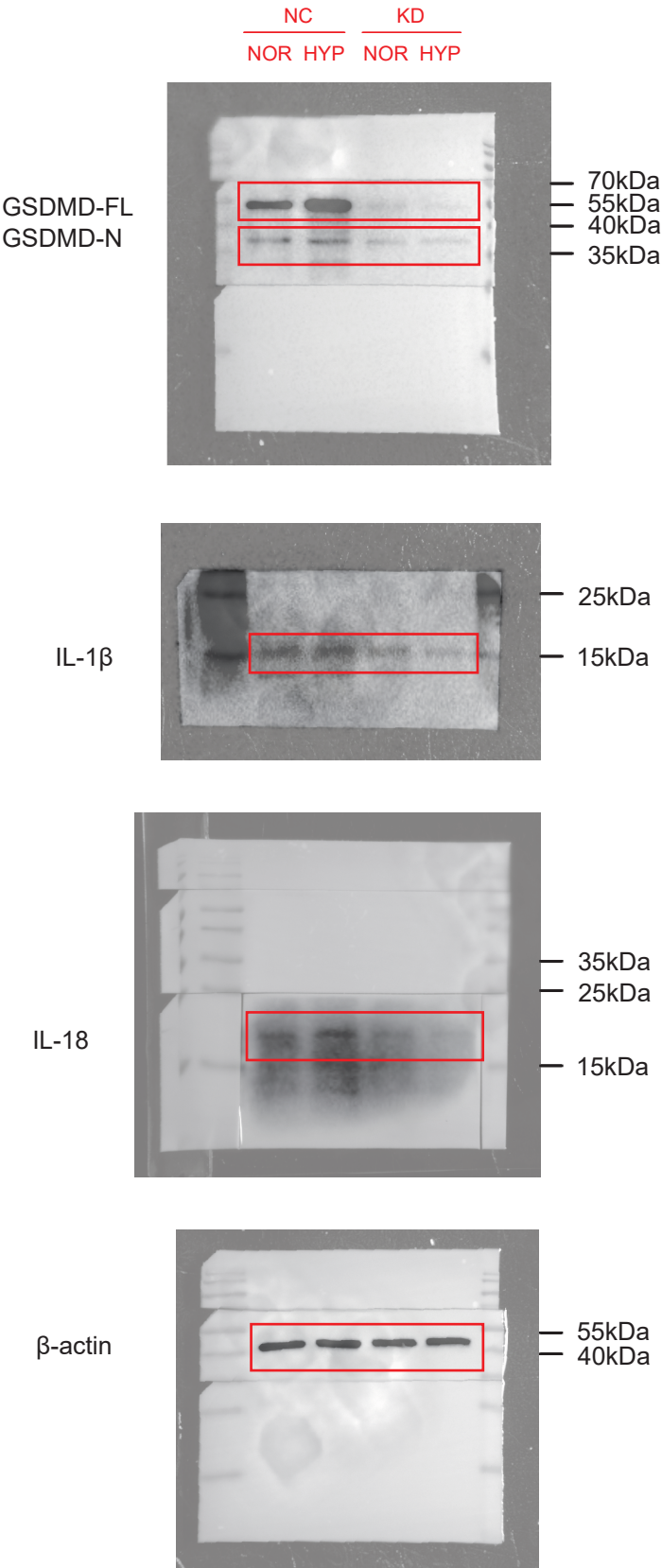

Figure 6D

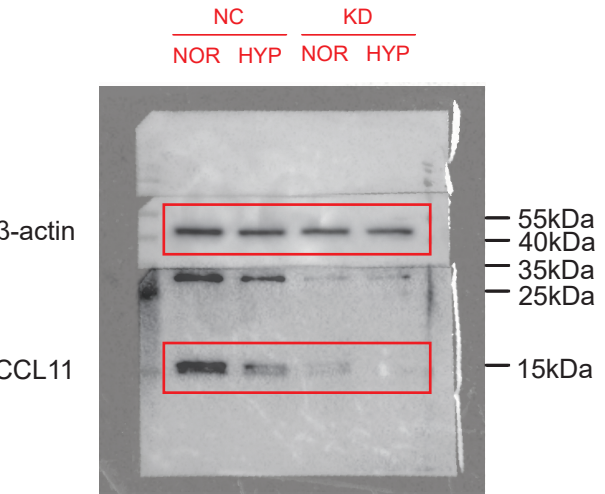

Supplement: Supplementary file 4 — Supplementary material 1 [file 41420_2023_1764_MOESM4_ESM.pdf]
